# Performance of the digital trail making test in older adults with white matter lesions

**DOI:** 10.3389/fnhum.2025.1572971

**Published:** 2025-07-23

**Authors:** Xinjun Shan, Le Tian, Hongyi Zhao, Xiuli Zhao, Fangyuan Wei

**Affiliations:** ^1^Department of Emergency, Baoding No. 1 Hospital, Baoding, China; ^2^Department of Neurology, Mental Health Institute of Inner Mongolia Autonomous Region, The Third Hospital of Inner Mongolia Autonomous Region, Hohhot, China; ^3^Department of Neurology, NO 984 Hospital of PLA, Beijing, China; ^4^Department of Neurology, The Seventh Medical Center of PLA General Hospital, Beijing, China; ^5^Department of Hand and Foot Surgery, Beijing University of Chinese Medicine Third Affiliated Hospital, Beijing, China

**Keywords:** cognitive dysfunction, fine motor control, white matter lesions, trail making test, small vessel disease

## Abstract

**Background:**

Studies have reported that digital assessment technology coupled with the traditional Trail Making Test (TMT) can capture additional information about a new cognitive domain.

**Objectives:**

The goal of the current study is to demonstrate the performance of the digital Trail Making Test (dTMT) in older people with white matter lesions (WML).

**Methods:**

In this single-center, observational study, 18 elderly patients with WML admitted to our hospital from June 2021 to June 2022 served as the WML group, and 18 participants matched for age, gender, and educational level who were undergoing physical examination in our hospital during the same period served as the control group. The participants completed the dTMT Part A (dTMTA) and dTMT Part B (dTMTB) to obtain the outcomes, such as dTMT completion time, number of errors, time inside each circle, total pathway deviation of each step, and velocity of drawing of each step. The severity of WML was scored using the Fazekas scale. Multiple neuropsychological assessments were carried out to assess cognitive function. The Purdue Pegboard Test (PPT) was used to display the unimanual dexterity (dominant hand task) and fine motor control (assembly task). The relationships between dTMT variables and cognition and motion in elderly patients with WML were analyzed using linear regression analysis.

**Results:**

The WML group required significantly more time to complete the dTMTA (19.78 ± 1.92 s vs. 18.17 ± 1.72 s, *p* = 0.012) and dTMTB (38.83 ± 4.33 vs. 34.00 ± 2.99, *p* < 0.001). For dTMTA, larger pathway deviation (43.76 ± 4.50 vs. 39.81 ± 4.66, *p* = 0.014) and lower velocity (17.05 ± 4.72 vs. 20.50 ± 4.00, *p* = 0.024) were found in the WML group. For dTMTB, longer time in the circle (30.02 ± 7.19 vs. 16.22 ± 4.70, *p* < 0.001), larger pathway deviation (47.00 ± 4.40 vs. 41.96 ± 6.44, *p* = 0.010), and lower velocity (11.48 ± 2.75 vs. 14.18 ± 3.86, *p* = 0.021) were exhibited in aged individuals with WML. Linear regression analysis showed that the time spent inside each circle showed a positive correlation with the Mini-Mental State Evaluation (MMSE) (*p* = 0.015, standardized *β* = 0.454) and PPT unimanual task (*p* = 0.024, standardized *β* = 0.420) for dTMTA; and the total time to completion was negatively correlated with the PPT assembly task (*p* = 0.001, standardized *β* = −0.583).

**Conclusion:**

Older adults with WML showed abnormalities while solving the dTMT. dTMT might be a potential indicator for cognitive and fine motor deficits in patients with WML.

## Introduction

With continued advances in medical technologies and improvement in life expectancy in modern society, cognitive impairment and motor disturbance have become common symptoms that negatively affect the daily life of the growing elderly population. In the last decade, an increasing number of studies have confirmed that cognitive impairment and motor dysfunction in older adults do not emerge in isolation (Zhào et al., 2023a). On the contrary, impairments in cognitive and physical dimensions are frequently concurrent (Fraser et al., 2010). Recent findings have even demonstrated the synergistic effects of cognitive and motor dysfunction in patients with cerebral small vessel disease (CSVD) (Jokinen et al., 2022).

White matter lesions (WML), along with cerebral microbleeds, recent subcortical lacunar infarcts (clinically symptomatic), lacunes (clinically silent), prominent perivascular spaces, and atrophy lacunar infarcts are known to be common signs of CSVD on conventional magnetic resonance imaging (MRI) (Rudilosso et al., 2022). WML represent a common condition in older adults, occurring in approximately 80% of adults in the general population over the age of 60 years (Moran et al., 2012). Cognitive dysfunction (especially executive dysfunction) is one of the main symptoms of WML (Siejka et al., 2018). Relatively little is known about changes in upper extremity motor function and control in patients with WML. In recent years, fine motor control has been evidenced to be another feature found in community dwellers with WML (Iandolo et al., 2024; Nyquist et al., 2015). However, the early detection of the above symptoms is difficult in clinical practice.

The traditionally conducted Trail Making Test (TMT) is a paper-and-pencil test and is considered a pure assessment tool for executive function, including processing speed, visuomotor skills, working memory, and cognitive flexibility (Sánchez-Cubillo et al., 2009). Recently, it has been reported that a well-designed digital Trail Making Test (dTMT) could be a useful tool for detecting cognitive impairment and fine motor control in patients with Parkinson’s disease (Park and Schott, 2022). Thus far, dTMT has exhibited the potential to discover both cognitive and motor domains concurrently (Libon et al., 2024). Previous research inferred that cognitive and motor timing ability is, to some extent, dependent on shared processing (Zhao H. et al., 2024). Thus, under dual-task conditions, the fine motor and/or cognitive task performance of older people can deteriorate due to competing demands when the available central resource capacity is exceeded (Piche et al., 2023). In contrast to ordinary dual-task tests that separate cognitive and motor tasks, the dTMT incorporates a cognitive task into handwriting (Wei et al., 2019). In addition, recently published findings have implied that cognitive tasks involving internal interfering factors (e.g., mental tracking) impair fine motor control more than those involving external interfering factors (e.g., reaction time) (Zhao H. Y. et al., 2024). Thus, the aim of the current study was to assess the performance of elderly individuals with WML using the dTMT task.

In the current study, we discovered that older adults with WML showed a lower level of abilities in dTMT Part A (dTMTA) and dTMT Part B (dTMTB), both of which displayed wider assessment domains beyond cognition.

## Materials and methods

### Participants

A total of 18 older adults with WML (WML group) and 18 healthy, age-matched individuals (HE group) were recruited from the Department of Neurology, the Seventh Medical Center of PLA General Hospital (which also serves older individuals in Aged Cadre Convalescent subdepartments). These patients were recruited consecutively from 1 June 2021 to 1 April 2022. The healthy aged individuals in the HE group, who had no history of WML diagnosis and who had regular rest and recuperation plans, were recruited from the Aged Cadre Convalescent subdepartment. Each participant voluntarily provided informed consent to participate in the current study. All participants underwent screening by 3.0 T MRI of the brain and were grouped based on a method previously described by our group (Zhao et al., 2024b). WML were graded using the Fazekas scale, as previously described (Zhào et al., 2023b). Briefly, we classified the severity of WML into three grades: grade 1 (“punctate lesions”) (*N* = 4), grade 2 (“early confluent lesions”) (*N* = 8), and grade 3 (“confluent lesions”) (*N* = 6). Individuals with a Fazekas score of 0 were classified into the HE group (*N* = 18). The exclusion criteria were as follows: the presence of a major stroke; other causes of leukoencephalopathy (including immune disorders, demyelination, and genetic conditions); major psychiatric disorders (diagnosed according to the DSM-IV criteria); use of psychotropic medications or drugs that increase the risk of falling (e.g., tranquillizers/sedatives, diuretics, and antiparkinsonian drugs); MRI contraindications; dementia (diagnosed with ICD-10); or a Mini-Mental State Evaluation (MMSE) score lower than 23 points.

Our study was approved by the Academic Ethics Committee of the Biological Sciences Division at the Seventh Medical Center of the PLA General Hospital in Beijing, China.

### MRI measurements

A 3.0 T MRI brain scan (Discovery MR750; GE Healthcare, USA) displayed WML, which indicated the degree of CSVD. Brain MRI (slice and interslice thicknesses of 5 mm and 1.5 mm, respectively) was carried out as follows: T1 fluid-attenuated inversion recovery (TR, 1750 ms; TE, 23 ms; TI, 780 ms; FOV, 24 cm) and T2-weighted imaging (TR, 7498 ms; TE, 105 ms; FOV, 24 cm) sequences.

### dTMT paradigm

The dTMT software was previously used as a feasible cognitive assessment tool for dementia screening (Wei et al., 2019). Briefly, a Windows Surface Pro 4 digitizer and a handheld stylus pen were used to assess drawing movements. This software was designed to analyze the drawing elements of the dTMT. The definitions of the drawing elements are shown in Table 1. The participants were given two tasks. In the first task, they were required to “draw a line as rapidly as possible joining consecutive numbers (i.e., 1- > 2- > 3… 9) in the circles randomly distributed on the screen.” This task was completed before dTMTA. For the second task, participants were instructed to “draw a line as rapidly as possible joining the numbers and Chinese characters (i.e.,1- > 壹- > 2- > 贰 … 玖) alternatively in the circles randomly distributed on the screen” before dTMTB. Examples of these tasks are shown in Supplementary Videos 1, 2.

**Table 1:** Clinical and demographic characteristics of the participants.

	WML group (*N* = 18)	HE group (*N* = 18)	*p*-value
Age, years	65.67 ± 19.31	64.17 ± 19.13	0.816
Men, %	9, 50.00	12, 66.67	0.190
Education, years	8.94 ± 3.31	9.89 ± 4.16	0.456
VFT, words	14.28 ± 6.06	15.78 ± 6.39	0.475
CDT, score	9.79 ± 1.17	10.00 ± 1.03	0.222
MMSE, score	28.00 ± 2.14	28.06 ± 1.66	0.931
PPT assembly task, assemblies	4.50 ± 1.15	5.18 ± 0.62	0.015*
PPT unimanual task, pegs	22.28 ± 2.14	23.89 ± 1.60	0.040*
dTMTA			
Time, s	19.78 ± 1.92	18.17 ± 1.72	0.012
Errors, times	0.61 ± 0.85	0.56 ± 0.78	0.084
Inside circles, s	8.09 ± 1.38	7.36 ± 1.50	0.139
Deviation, mm	43.76 ± 4.50	39.81 ± 4.66	0.014*
Velocity, mm/s	17.05 ± 4.72	20.50 ± 4.00	0.024*
dTMTB			
Time, s	38.83 ± 4.33	34.00 ± 2.99	0.001**
Errors, times	2.392 ± 1.38	1.72 ± 10.96	0.101
Inside circles, s	30.03 ± 7.19	16.22 ± 4.70	0.001**
Deviation, mm	47.00 ± 4.40	41.96 ± 6.44	0.010*
Velocity, mm/s	11.48 ± 2.75	14.18 ± 3.86	0.021*

(mean ± SD), except for Male, %.

*p < 0.05, **p < 0.01.

VFT, verbal fluency test; CDT, clock drawing test; MMSE, Mini-Mental State Examination; PPT, Purdue Pegboard test; dTMT, digital Trail Making Test; WML, white matter lesions; HE, healthy; s, seconds; ms, milliseconds; mm, millimeters.

Previous studies have suggested that the time taken to completion on both dTMTA and dTMTB assesses overlapping yet different underlying neurocognitive constructs (Recker and Poth, 2023; Saalfield et al., 2024). TMT Part A (TMTA) measures visual search and processing speed; TMT Part B (TMTB) measures working memory and, secondarily, task-switching ability (Sánchez-Cubillo et al., 2009).

### Neuropsychological assessments

All participants completed a series of neuropsychological assessments, including MMSE (reflecting global cognitive level), the choice reaction test (CRT) (reflecting attention and concentration), the digit symbol substitution test (DSST) (reflecting visuoperceptual functions and processing speed), the category verbal fluency test (VFT) (reflecting psychomotor speed and semantic memory), and the clock drawing test (CDT) (reflecting visuospatial function). Details about these assessments are described in our previous studies (Zhào et al., 2021a).

### Manual dexterity evaluation

Unimanual dexterity was evaluated using the Purdue Pegboard test (PPT) unimanual task (UPC 32027; Lafayette Instruments, Lafayette, IN). Each participant was instructed to use the dominant hand to place as many pegs as possible into the holes within 30 s (Sanal-Hayes et al., 2025).

Instructions for dominant and non-dominant hand are as follows: “Pick up one pin at a time with your right hand/left hand (depending on dominant hand) from the right-handed/left-handed cup (if right-handed then right cup). Starting with the top hole, place each pin in the right hand/left hand row (if right-handed then right-hand row). Now you may insert a few pins for practice. If during the testing time you drop a pin, do not stop to pick it up. Simply continue by picking another pin out of the cup.”

Fine motor control was evaluated using the PPT assembly task (Chen et al., 2024). Each participant was instructed to assemble small objects (washers and collars) within 60 s (Sanal-Hayes et al., 2025).

Instructions for assembly for right-handed people are as follows: “Pick up one pin from the right-hand cup with your right hand. While you are placing it in the top hole in the right-hand row, pick up a washer with your left hand. As soon as the pin has been placed, drop the washer over the pin. While the washer is being placed over the pin with your left hand, pick up a collar with your right hand. While the collar is being dropped over the pin, pick up another washer with your left hand and drop it over the collar. This completes the first ‘assembly,’ consisting of a pin, a washer, a collar, and a washer. While the final washer for the first assembly is being placed with your left hand, start the second assembly immediately by picking up another pin with your right hand. Place it in the next hole, drop a washer over it with your left hand, and so on, completing another assembly. Now, take a moment to try a few practice assemblies.”

### Definitions for dTMT variables

All the definitions for dTMT variables are provided as previously described by Wei et al. (2019). Brief descriptions are listed as follows: The total time to completion (time): the time taken (s) to draw a line connecting all circles in the correct order. The number of errors (errors): the number of times a line is drawn to a circle in the incorrect order. The time inside circles (inside circles): the time spent in (s) to draw inside circles. The pathway deviation of each step (deviation): the actual line in (mm) minus the nearest line in (mm) of each step. The velocity of drawing of each step (velocity): the actual line in (ms) of each step divided by the time (s) to completion for each step.

### Statistical analysis

Student’s *t*-test was carried out to compare continuous parametric variables. Linear regression analysis was performed to investigate the correlation between dTMT variables and cognitive function/fine motor control, adjusted for age, gender, and education level. Considering that age, gender, and education level were potential confounding factors, model 1 was linear regression analysis, and model 2 was linear regression analysis with adjustment. Pearson’s correlation analysis of all data from both groups was used to demonstrate the relationship between the cognitive/fine-motor function and Fazekas scale severity level. The significance threshold was set at *p* < 0.05. Analysis was carried out using SPSS 22.0 software.

## Results

As shown in Table 1, age, sex, and level of education score were similar between the WML and HE groups (*p* > 0.05). Overall, the WML group performed significantly worse on the dTMTA (19.78 ± 1.92 s vs. 18.17 ± 1.72 s, *p* = 0.012) and dTMTB (38.83 ± 4.33 s vs. 34.00 ± 2.99 s, P < 0.001) compared to the HE group. However, there was no obvious difference in scores obtained from other cognitive tests. The individuals in the WML group exhibited a significantly lower level of fine motor control during the 60s PPT assembly task (WML group = 4.50 ± 1.15 u vs. HE group = 5.18 ± 0.62 u assemblies, *p* = 0.015). Additionally, unimanual dexterity was marginally affected, as indicated by the results from the 30s PPT unimanual task (WML group = 22.28 ± 2.14 u vs. HE group = 23.89 ± 1.60 pegs, *p* = 0.040). The participants in the WML group had an average Fazekas score of 2.11. As shown in Supplementary Table 1, there existed a close relationship between the PPT unimanual task and the Fazekas scale severity level (*r* = −0.441, *p* = 0.007).

When inspecting the dTMTA variables, the aged adults with WML displayed larger pathway deviation (WML group = 43.76 ± 4.50 u vs. HE group = 39.80 ± 4.66 u mm, *p* = 0.014) and lower velocity (WML group = 17.05 ± 4.72 u vs. HE group = 20.50 ± 4.00 u mm/s, *p* = 0.024). When inspecting the dTMTB variables, the patients with WML showed longer time in circle (WML group = 30.03 ± 7.19 u vs. HE group = 16.22 ± 4.70 u s, *p* < 0.001), larger pathway deviation (WML group = 47.00 ± 4.40 u vs. HE group = 40.96 ± 6.43 u mm, *p* = 0.010), and lower velocity (WML group = 11.48 ± 2.75 u vs. HE group = 14.17 ± 3.86 u mm/s, *p* = 0.021).

Furthermore, the association between dTMT variables and cognitive function/fine motor control was explored using linear regression analysis adjusted for age, gender, and education level. When inspecting the dTMTA variables, the time inside each circle was positively correlated with MMSE (*p* = 0.007, standardized *β* = 0.397) and the PPT unimanual task (*p* = 0.010, standardized *β* = 0.446). The pathway deviation of each step was negatively correlated with the PPT assembly task (*p* = 0.037, standardized *β* = −0.393). When inspecting the dTMTB variables, the total time to completion was negatively correlated with the PPT assembly task (*p* = 0.003, standardized *β* = −0.522). The trends were still found after adjusting for age, gender, and educational level. Details are shown in Tables 2, 3 and Supplementary Figure 1. Several dTMT variables were found to be associated with fine motor control during the test.

**Table 2:** Association between dTMTA variables and cognitive/fine motor control performance.

	Time	Errors	Inside circles	Deviation	Velocity
	Standardized *β*, *p*-value	Standardized *β*, *p*-value	Standardized *β*, *p*-value	Standardized *β*, *p*-value	Standardized *β*, *p*-value
VFT					
Model 1	−0.001, 0.996	0.148, 0.544	−0.126, 0.549	−0.251, 0.301	0.091, 0.772
Model 2	0.233, 0.089	0.163, 0.578	−0.190, 0.450	−0.286, 0.288	0.092, 0.762
CDT					
Model 1	−0.069, 0.753	0.234, 0.274	−0.043, 0.813	0.013, 0.952	−0.061, 0.786
Model 2	−0.070, 0.750	0.247, 0.281	−0.054, 0.782	−0.015, 0.942	−0.047, 0.841
MMSE					
Model 1	−0.012, 0.953		−0.012, 0.953		−0.012, 0.953
Model 2	−0.015, 0.939		−0.015, 0.939		−0.015, 0.939
PPT unimanual task					
Model 1	−0.230, 0.243	0.266, 0.163	0.446, 0.010*	−0.009, 0.963	−0.192, 0.337
Model 2	−0.219, 0.275	0.264, 0.207	0.420, 0.024*	−0.125, 0.508	0.138, 0.523
PPT assembly task					
Model 1	−0.232, 0.227	−0.036, 0.843	0.219, 0.172	−0.393, 0.037*	−0.006, 0.975
Model 2	−0.324, 0.105	−0.050, 0.807	0.241, 0.174	−0.426, 0.028*	0.015, 0.942

Model 1 represents the relation without adjustment; Model 2 represents the relation adjusted for age, gender, and educational level.

*p < 0.05, **p < 0.01.

VFT, verbal fluency test; CDT, clock drawing test; MMSE, Mini-Mental State Examination; PPT, Purdue Pegboard test.

**Table 3:** Association between dTMTB variables and cognitive/fine motor control performance.

	Time	Errors	Inside circles	Deviation	Velocity
	Standardized *β*, *p*-value	Standardized *β*, *p*-value	Standardized *β*, *p*-value	Standardized *β*, *p*-value	Standardized *β*, *p*-value
VFT					
Model 1	−0.373, 0.097	−0.084, 0.718	−0.362, 0.115	0.096, 0.696	0.470, 0.055
Model 2	−0.296, 0.218	0.082, 0.752	−0.409, 0.132	0.185, 0.513	0.585, 0.039*
CDT					
Model 1	0.195, 0.312	0.344, 0.096	0.356, 0.077	−0.109, 0.613	−0.013, 0.951
Model 2	0.280, 0.137	0.327, 0.112	0.378, 0.076	−0.064, 0.770	0.042, 0.843
MMSE					
Model 1	−0.020, 0.907	0.201, 0.269	0.073, 0.678	−0.247, 0.203	0.047, 0.800
Model 2	0.073, 0.661	0.179, 0.328	0.097, 0.604	−0.195, 0.329	0.109, 0.568
PPT unimanual task					
Model 1	0.077, 0.649	0.131, 0.466	0.283, 0.111	0.116, 0.544	0.184, 0.322
Model 2	0.095, 0.573	0.106, 0.563	0.268, 0.161	0.211, 0.296	0.266, 0.175
PPT assembly task					
Model 1	−0.522, 0.003**	−0.252, 0.156	−0.310, 0.075	−0.173, 0.354	0.168, 0.351
Model 2	−0.583, 0.001**	−0.326, 0.079	−0.308, 0.10	−0.183, 0.356	0.137, 0.470

Model 1 represents the relation without adjustment; Model 2 represents the relation adjusted for age, gender, and educational level.

*p < 0.05, **p < 0.01.

VFT, verbal fluency test; CDT, clock drawing test; MMSE, Mini-Mental State Examination; PPT, Purdue Pegboard test.

## Discussion

In the current preliminary study, we developed a digitized version of TMT, and managed to compare the dTMT variables in aged adults with or without WML. dTMT expands on the scope of evaluation of traditional TMTby incorporating many dynamic parameters, including time, errors, inside circles, deviation, and velocity. The completeness of patients with WML on the dTMT was found to be poorer compared to aged healthy individuals. First, the total completion time of both dTMTA and dTMTB differed between groups. Second, aged adults with WMLs showed a larger pathway deviation and lower velocity during dTMTA, while for dTMTB, they needed a longer time inside each circle.

TMT is commonly used in neuropsychological assessments, reflecting the executive function in CSVD and other neurological disorders (Fellows et al., 2017). Due to the high sensitivity and specificity, TMT was considered as one of the “Minimum-battery of Tests” to detect mobility and cognition in aged adults with neurodegenerative diseases (Montero-Odasso et al., 2019). Along with the development of digitized technology, several researchers tried to identify novel aspects of motor and cognitive function than the traditional version (Libon et al., 2024; Park and Schott, 2022). Our preliminary study once again demonstrated that dTMT based on simplified Chinese characters could be used to find several aspects of cognitive and fine-motor decline in aged patients with WML. In line with these findings, our previous work also demonstrated that modifying the traditional clock drawing test into a digital version could bring about a new domain of cognition in aged individuals with WML (Zhào et al., 2019). For dTMTA, pathway deviation and drawing velocity differed between groups, whereas, for dTMTB, the differences between groups existed in pathway deviation, drawing velocity, andtime in circle. Although TMT was invented as a cognitive assessment, dTMT can be used as a motor-cognitive dual task (Herold et al., 2018). In more detail, motor variables such as drawing velocity were collected during the fulfillment of the cognitive task. As has been evidenced in our previous works, poor dual-task ability was characterized as a typical WML symptom (Ma et al., 2022). This point might be the rational reason why variables collected from dTMTB were more obvious than those from dTMTA. As we know, completing dTMTB needs more cognitive reservation compared to dTMTA, since the former reflected processing speed and more complex cognitive abilities, while the latter reflected only visual search speed and tracking (Fellows et al., 2017).

In contrast to the discovery of cognitive dysfunction in CSVD, the findings around fine-motor abnormalities in aged adults with WML were limited. By using a handwriting analysis system, our previous studies have confirmed that patients with WML exhibited fine-motor disorder, which is a tiny but typical symptom associated with disease severity (Wei et al., 2019; Zhào et al., 2019, 2021b, 2023b). All these results were along with the findings reported by Iandolo et al. (2024), Nyquist et al. (2015), and Sachdev et al. (2005) using pegboards. In the present study, we showed once again that fine motor control was abnormal in aged patients with WML. Besides, this phenomenon could be detected by dTMT. Fine motor control, as reflected in the PPT assembly task, was found to be associated with both dTMTA (the time inside circles) and dTMTB (the total time to completion).

Several limitations of the current study merit consideration. First, the sample size was small. Second, dTMT was designed as a modified version, with only 9 circles located in dTMTA and 18 circles located in dTMTB.

In the present preliminary study, older adults with WML demonstrated poorer dTMT performance compared to healthy control participants. The dTMT was associated with aspects of cognitive function fine motor control. Therefore, solving the dTMT might represent a potential indicator for early identification of the cognitive and mobility decline induced by WML. With the development of digitized technologies, besides dTMT, more traditional cognitive tests such as digital CDT and digital Rey–Osterrieth Complex Figure (Zhang et al., 2021) could be modified to detect cognitive deficits and motor abnormalities in patients with WML in future.

## Data Availability

The datasets presented in this study can be found in online repositories. The names of the repository/repositories and accession number(s) can be found in the article/Supplementary material.
