# Supplementary material for: Performance of the digital trail making test in older adults with white matter lesions
Source: Front Hum Neurosci. 2025 Jul 23;19:1572971. doi: 10.3389/fnhum.2025.1572971 (PMC12325341; doi:10.3389/fnhum.2025.1572971)
Supplement: Supplementary file 1 [file Table_1.docx]

**Supplementary Table 1 Pearson correlation between cognitive/fine-motor function and** **Fazekas scale severity level.**

|  | Fazekas scale severity level | |
| --- | --- | --- |
|  | **r value** | **P value** |
| VFT, score | -0.104 | 0.546 |
| CDT, score | -0.154 | 0.370 |
| MMSE, score | 0.008 | 0.962 |
| PPT assembly task, assemblies | -0.314 | 0.062 |
| PPT unimanual task, pegs | -0.441 | 0.007** |

**P ＜ 0.01

MMSE: Mini-Mental State Examination; VFT: Verbal Fluency Test; CDT: Clock Drawing Test; PPT: Perdue Pegboard Test
